# Parallel effects of memory set activation and search on timing and working memory capacity

**DOI:** 10.3389/fpsyg.2014.00779

**Published:** 2014-07-28

**Authors:** Richard Schweickert, Claudette Fortin, Zhuangzhuang Xi, Charles Viau-Quesnel

**Affiliations:** ^1^Department of Psychological Sciences, Purdue UniversityWest Lafayette, IN, USA; ^2^École de Psychologie, Université LavalQuébec, QC, Canada

**Keywords:** time production, working memory capacity, complex memory span, activation, retrieval, memory search, additive factor method, selective influence

## Abstract

Accurately estimating a time interval is required in everyday activities such as driving or cooking. Estimating time is relatively easy, provided a person attends to it. But a brief shift of attention to another task usually interferes with timing. Most processes carried out concurrently with timing interfere with it. Curiously, some do not. Literature on a few processes suggests a general proposition, the Timing and Complex-Span Hypothesis: A process interferes with concurrent timing if and only if process performance is related to complex span. Complex-span is the number of items correctly recalled in order, when each item presented for study is followed by a brief activity. Literature on task switching, visual search, memory search, word generation and mental time travel supports the hypothesis. Previous work found that another process, activation of a memory set in long term memory, is not related to complex-span. If the Timing and Complex-Span Hypothesis is true, activation should not interfere with concurrent timing in dual-task conditions. We tested such activation in single-task memory search task conditions and in dual-task conditions where memory search was executed with concurrent timing. In Experiment 1, activating a memory set increased reaction time, with no significant effect on time production. In Experiment 2, set size and memory set activation were manipulated. Activation and set size had a puzzling interaction for time productions, perhaps due to difficult conditions, leading us to use a related but easier task in Experiment 3. In Experiment 3 increasing set size lengthened time production, but memory activation had no significant effect. Results here and in previous literature on the whole support the Timing and Complex-Span Hypotheses. Results also support a sequential organization of activation and search of memory. This organization predicts activation and set size have additive effects on reaction time and multiplicative effects on percent correct, which was found.

## Introduction

Accurately estimating a brief time interval is important in numerous everyday activities including talking, playing music and performing in sports. In studying timing performance, people are often asked to reproduce a short time interval by tapping a finger twice. This is relatively easy, provided a person attends to it. A brief shift of attention to another task usually interferes with timing, however. According to a prevalent accumulation model, timing demands are limited and well defined: Pulses are generated by an internal pacemaker, a gate allows pulses to be sent to an accumulator, and when the pulse count reaches a criterion, a movement ending the temporal reproduction is prompted. Attention controls the gate, the criterion and accumulated pulses require memory storage, and comparing accumulated pulses with the criterion requires attention (Gibbon et al., 1984; Zakay and Block, 1996; Brown, 2006; Buhusi and Meck, 2009). For recent reviews see Buhusi and Meck (2005, 2009). Timing is sensitive to the relentless attention and memory requirements throughout the reproduced interval, making timing a sensitive indicator of demands in secondary tasks.

Timing is also likely to be sensitive to demands of ongoing internal processing, thinking, mind-wandering, and so on. Indeed, there is evidence that mental time travel interferes with timing (El Haj et al., 2013). As words or images arise internally during production of a time interval, they compete for resources allocated to timing. Further, they occasionally produce a cue or prime that by association activates information in secondary memory. Does mere activation of information interfere with timing, or does activated information interfere only if it is used? Internal cues are difficult to control experimentally, of course. Here we address the corresponding questions with regard to a secondary task.

Brown (1997) reported that many secondary tasks interfere with concurrent timing. But not all do. High on the list of candidates likely to influence concurrent timing, in Brown's view, are executive processes such as coordination and scheduling, because they demand attention and working memory. As an example, Brown (2006) showed that a particular executive process, random number generation, interfered with concurrent timing. But even among executive processes, some interfere with timing and some do not. For example, Fortin et al. (2010) showed that task switching, an executive process, did not.

A difficulty in ascertaining which processes interfere with timing is that the term “working memory” is broad. A way forward is provided by a well-specified measure of working memory capacity, complex span (Daneman and Carpenter, 1980). People with high complex span are more accurate at timing than those with low complex span (Broadway and Engle, 2011).

In a complex memory span task, a person performs an activity (such as subtraction), stores an item; performs another activity and stores another item, continuing until the sequence of activities and items is finished. Finally, items are recalled in order. The score is typically the number of items correctly recalled in correct serial positions (e.g., Unsworth and Engle, 2007, p. 110). Individuals with high complex-span perform better on various tasks than individuals with low complex-span. The executive attention view of working memory capacity (Engle and Kane, 2004; Kane et al., 2007) explains this by saying high-working memory capacity individuals have better ability to maintain goals. In a complex-span task it is important to maintain the goal of remembering items while carrying out an unrelated activity such as subtraction. For recent discussion of this view and of tasks related to working memory capacity, see Unsworth et al. (2012). This view is remarkably similar to the explanation that timing requires continual maintaining of the goal to keep time, and is interfered with by tasks that distract from the goal. With this view, when timing and a secondary task are done together, if interference occurs, it is the result of some particular secondary task process distracting from the goal of timing.

When we consider the few tasks whose effect on timing and whose relation to complex-span are both known, there are striking parallels. (a) Task switching does not interfere with timing (Fortin et al., 2010) and task switching performance is not related to complex-span (Kane et al., 2003). (b) The same is true for attention-demanding visual search (for timing, Fortin et al., 1993; Schweickert et al., 2007; for complex span, Kane et al., 2006). (c) Sternberg memory search interferes with concurrent timing (e.g., Fortin and Rousseau, 1987) and performance is related to complex-span (Conway and Engle, 1994). (d) Generating words starting with a given letter increases variance in time production (Ogden et al., 2011) and performance is related to complex-span (Unsworth et al., 2011; see also Rosen and Engle, 1997). We tentatively add a fifth, internal process. (e) Mental time travel is related to timing (El Haj et al., 2013) and is related to complex-span (Mrazek et al., 2012). The last statement is tentative because the timing experiment by El Haj et al. (2013) differs considerably from the others mentioned. Participants verbally estimated durations longer than 30 s in prospective and retrospective timing paradigms rather than producing intervals shorter than 5 s in a prospective paradigm. Further, evidence of mental time travel is indirect. It was inferred by El Haj et al. (2013) from Remember/Know judgments in a recognition task. Evidence was indirect also in Mrazek et al. (2012). They presented thought sampling probes while participants performed complex-span tasks; performance was negatively correlated with amount of attention to task unrelated concerns, much of which is likely to be mental time travel (Corballis, 2012). Reviews of timing tasks are in Brown (1997, 2006) and Fortin (1999). For tasks whose performance correlates well with Working Memory Capacity, see the review by Kane et al. (2007, p. 35).

In these examples complex-span is unrelated to processes that do not interfere with timing (task switching and visual search) but related to processes that do so (memory search, word generation and possibly, mental time travel). A generalization from these examples is that timing and a process executed concurrently with it interfere if and only if performance of the process varies with complex memory span. We call this the Timing and Complex-Span Hypothesis. If true the hypothesis tightens the previous characterization of processes interfering with timing as those that are executive. Of course, interference and variation are matters of degree. A more precise statement of the hypothesis is that a process interferes with concurrent timing to the degree that process performance varies with complex memory span. Because the literature typically classifies processes as interfering with timing or not, or as related to complex span or not, we discuss the hypothesis here in a dichotomous form.

A test immediately arises from the paper by Conway and Engle (1994). They examined two processes, short term memory search and activation (retrieval) of items to be searched. Memory search satisfies the proposition. Performance on activation was the same for low and high complex-span individuals. If the Timing and Complex-Span Hypothesis is true, activating a set of items to be searched will not interfere with concurrent timing. Activating a memory set is particularly interesting because it is an important component of complex memory span tasks. In the steps of a complex memory span trial listed above, is the penultimate step, activating items in long-term memory, a source of interference with timing?

Experiments here address whether activating a memory set interferes with timing; work of Conway and Engle (1994) already establishes activation is not related to complex span. In the Sternberg (1966) memory search task, a participant memorizes a short list, the memory set. A probe is then presented and the participant indicates whether or not the probe was present in the memory set. When a memory set has been learned so well that it is in long-term memory, but because of decay or interference is no longer in short-term memory, it must be activated before it can be searched (Wickens et al., 1981, 1985; Conway and Engle, 1994). The need for activation increases reaction time. Whether activation will affect concurrent timing or not is difficult to predict a priori because activation borders a process that interferes with timing (memory search) and a processes which does not (task switching). On the one hand, activating a memory set would seem to use some of the same resources as searching a memory set, which interferes consistently with time production (Fortin and Rousseau, 1987; Fortin et al., 2007; Rattat, 2010). On the other hand, activating a memory set when multiple sets have been learned is a switch from one memory set to another. Although there is a cost to switching memory sets (Humphreys et al., 2009), switching between digit classification and memory search did not interfere with time production (Fortin et al., 2010). Furthermore, activation is an automatic process in some theories (e.g., Anderson, 1983) and as such, should not interfere with other concurrent processes. Depending on what resources activation shares with memory search or with switching, activating a memory set would interfere or not with concurrent timing.

### Process organization

**Figure 1:**
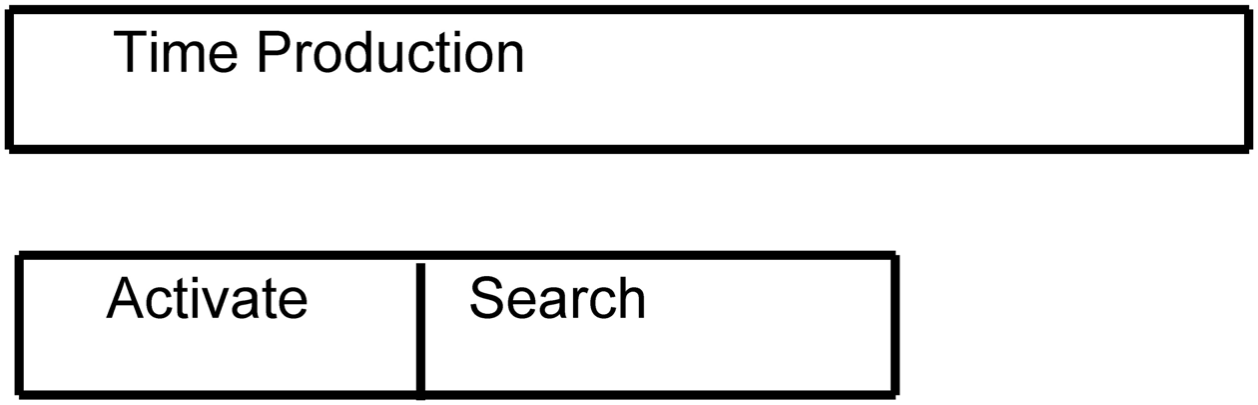
**In the Dual Task Condition, a tone and probe are presented simultaneously**. Time production and the memory task begin simultaneously (left side of figure). In the process organization proposed by Wickens et al. (1981), the first memory-task process is activation of the memory set. When it finishes, memory search begins. When the target time interval has elapsed and the memory task is finished, the response is made (right side of figure). In the Dual-Task Condition, time production is concurrent with activation and search. The time production process is not present in the Single-Task Condition.

We are also interested in memory set activation for a reason not directly related to what we have said so far, to consider predictions of a model of process organization originally proposed by Wickens et al. (1981), later used by Wickens et al. (1985) and Conway and Engle (1994). According to the model activating and searching a memory set are carried out successively (see the lower part of Figure 1). Wickens et al. (1981) gave participants a list to memorize, of size 2 or 4. They then presented a probe to search for in the list, either immediately or after an interval of counting backwards by threes. If the probe was presented after the interval, reaction time increased, attributed to the time needed to activate the memory set. When set size increased, reaction time also increased, explained by a longer time needed to search a larger memory set. The combined effect on reaction time of presenting the probe after an interval and of increasing set size was the sum of their individual effects. Such additivity was also found by Wickens et al. (1985). Additivity is explained if the participant first activates the memory set and then searches it. The time to complete the task is the sum of the durations of each process, so the combined effect of prolonging both processes is the sum of the individual effects (Sternberg, 1969).

In related experiments, Conway and Engle (1994) asked participants to memorize sets of different sizes. Then, on each trial, a cue was presented to indicate which memory set was relevant on the trial, followed by a probe to search for in the memory set. On some trials there was a delay between the cue and the probe, allowing time to activate the cued memory set. Conway and Engle (1994) found additive effects on reaction time of absence of the delay and of memory set size (set size 2 was sometimes an exception). Additivity is explained as by Wickens et al. (1981): the participant first activates the memory set and then searches it. Such sequential organization, if it occurs, separates an effect of activation difficulty from an effect of search difficulty, facilitating an answer to the question of whether activation interferes with timing. If memory set activation and search are in series, and both are concurrent with time production, they are organized as in Figure 1. The organization is similar to that proposed for time production concurrent with a visual search task by Schweickert et al. (2007).

The primary issue here is whether activating information from long-term memory interferes with concurrent timing. Testing activation and search in memory allowed us to examine the secondary issue, whether activating and searching a memory set are executed successively or not. To study these issues, activating and searching a memory set were performed in two main conditions. In the Single-Task Condition the participant performed the search task alone, and reaction time was the main dependent measure. In the Dual-Task Condition, the participant performed the search task while concurrently producing a time interval, and time production was the main dependent measure. Errors in memory search were also analyzed. In both conditions, the participant sometimes had to activate the memory set. The Single-Task Condition allowed us to determine whether the need for activation increased the time to perform the task. The Dual-Task Condition allowed us to determine whether the need for activation interfered with timing. To explore the generality of results of Conway and Engle (1994), the first two experiments used a paradigm somewhat different from theirs. Participants memorized two short lists to a high criterion, so the lists were in secondary memory. To ensure that one particular list was always in the activated state, it was presented again at the start of each trial. Experiment 3 used the paradigm of Conway and Engle (1994).

## Experiment 1

### Materials and methods

Trials and blocks of trials had the same basic structure in Experiments 1 and 2. In both experiments, the participant memorized two sets, one of words and one of letters, at the beginning of each block of trials. Words were from the pool {BIB, CAR, CUB, DAM, DOG, HAT, HIP, KIT, KEG, MAN, MUD, PEN, PIT, RUG, SOD, TAB, TIN, WAX, WIG, ZIT}. The letter pool was the 20 consonants (excluding Y). One pool and the memory set formed from it had already been selected by the experimenter to be called active, the other pool and set formed from it to be called inactive.

**Figure 2:**
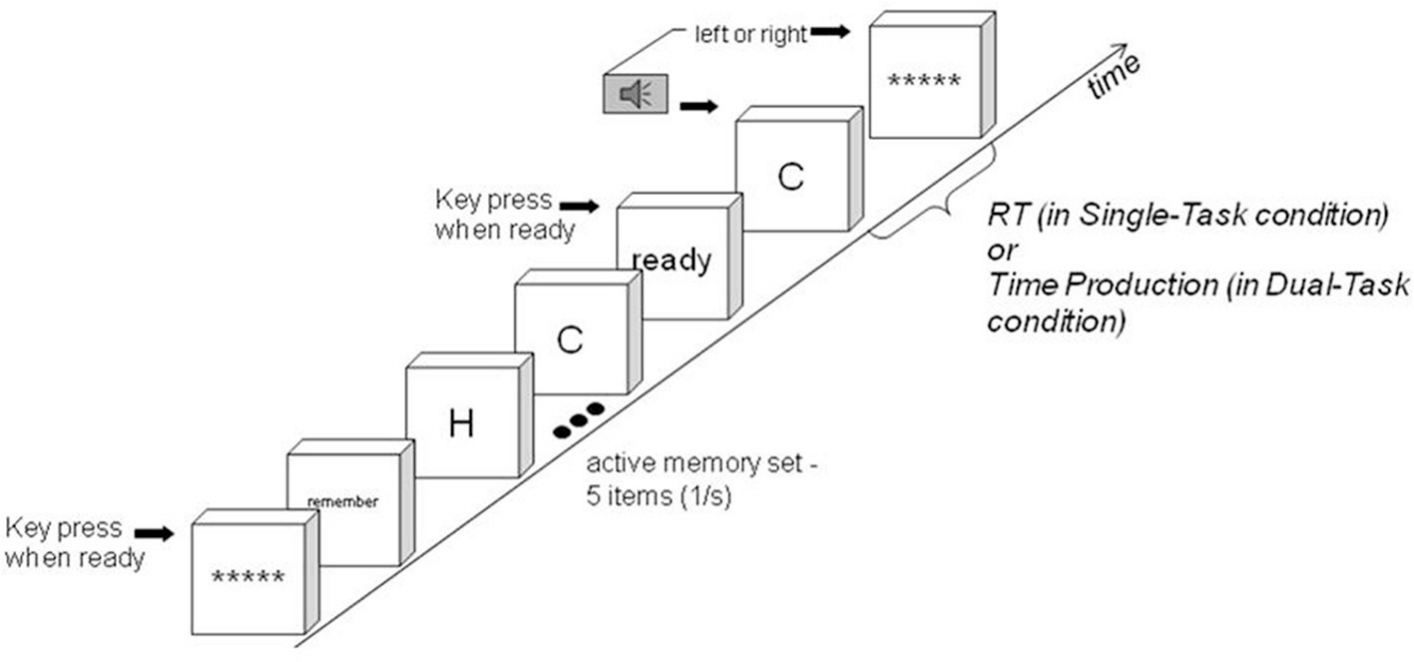
**A trial in the search phase**. *Single-Task Condition:* The interval between the probe onset and the left/right key press response is the Reaction Time. *Dual-Task Condition:* The trial is identical except the participant must attend to the tone's duration: the presence or absence response is to be given only when the tone has reached the subjective target duration. The interval between the probe onset and the left/right key press response is the Time Production.

At the beginning of each trial, the active set was presented again (see Figure 2, where the letter set is active). The inactive set was never presented after it was memorized. Then a probe was presented: a word or a letter. The task was to indicate whether the probe was present in either memory set or absent from both. (Logically, the probe could be present in at most one memory set). If a probe was from the active pool, the participant could search for the probe in immediate memory. But if the probe was from the inactive pool, the inactive set presented at the beginning of the block had to be activated before being searched. The pool the probe was from determined whether or not activation from long term memory was needed on a trial.

In Experiment 1, two factors were varied on trials: presence or absence of the probe in the memory set and whether the probe was from the active or inactive memory pool. Memory search was performed in a Single-Task Condition and in a Dual-Task Condition. A different group of participants was tested in each condition to avoid carry-over from one mode of responding to another.

#### Single-task condition

***Participants.*** Fourteen Purdue University undergraduates participated to partially fulfill an introductory psychology course requirement. Each was run individually in four sessions. The cutoff of 0.25 was the maximum error proportion allowed in each of the four factor combinations (probe present/absent and memory set active/inactive), averaged over all four sessions. Four participants exceeded this cutoff so their data were eliminated. (The memory task was more difficult than expected for the participants, as will be discussed later). Ages of the ten participants whose data were used (two men, eight women) ranged from 18 to 24 years (*M* = 19.9; *SD* = 1.85). Approval for human participants was obtained from the Purdue University Institutional Review Board. The experiment conforms to relevant regulatory standards. Informed consent was obtained from all participants.

***Stimuli and apparatus.*** The experiment was controlled by E-Prime (1.1). Participants removed watches. They sat 60 cm in front of a computer screen. Reaction times and time productions were timed to the nearest millisecond. Responses were made with a button box (Psychology Software Tools), with the three leftmost buttons denoted left, middle and right respectively. Index, middle and ring fingers of the right hand rested on those buttons.

Letter sets consisted of five items selected randomly without replacement from the letter pool. Word sets were formed likewise, of size five. All stimuli were white on black background, Courier New, font size 18 pts.

***Design and procedure.*** In each session six test blocks followed a practice block of trials. Each block had 25 trials. Participants were not told about the practice.

At the start of the learning phase of a block, a letter memory set and a word memory set appeared on the screen, one set at a time. Different sets were used in each block. On the first (practice) block, the set presented first was chosen at random. On half of the six test blocks, at random, the letter set appeared first; on the other half the word set appeared first. The five items of a memory set were presented in a vertical column, the first item (at the top) centered on the screen. The participant memorized the first memory set. Then the participant turned his or her head away from the screen and recited the items in order (top to bottom). The experimenter determined whether the set was recalled correctly. If recall was incorrect, the participant studied the set again. Recall was correct when all items were recited in order with no intrusions. When recall was correct, the participant pressed the middle button to display the next memory set. The participant memorized the second set and was tested as for the first. When finished, the participant pressed the middle button to start the search phase of the block.

In the search phase of a block, one of the two pools was chosen randomly to be active throughout the block. In three of the six test blocks, the letter pool was active. Each trial began with presentation of five asterisks in a row centered on the screen, indicating the participant could start the trial by pressing the middle button. When the middle button was pressed, “Remember” appeared, followed by the five items of the active memory set, one at a time, centered on screen. “Remember” and each item were presented for one second, with no delay between them. The last item was followed immediately by “Ready.”

When ready, the participant pressed the middle button, starting a tone presentation. The probe (“C” in Figure 2) appeared when the tone began. The participant was instructed to ignore the tone (it was relevant in the Dual-Task Condition only). The participant then pressed the left button if the probe was present in either memory set and the right button if it was absent, under instructions to respond as quickly as possible. When the response was made, the tone ended and a row of five asterisks appeared on the screen, indicating a new trial starting.

For a given participant in a given session, probes were selected as follows. On the first trial of the practice block, the probe was randomly selected from one of the pools and was randomly present or absent. On the first trial of a test block, the probe was present on half the trials, at random. The first trial was not analyzed. On half of the 24 trials following the first, the probe was from the active memory pool and on half from the inactive memory pool, at random. On half of the 12 trials with the probe from the active pool, the probe was present in the active memory set and on half it was absent, at random. The same applied for the other 12 trials with the probe from the inactive pool.

Blocks of trials were separated by a 30 s pause. Each session lasted approximately 45 min. Two sessions were never on the same day or more than a week apart.

#### Dual-task condition

***Participants.*** Fifteen Purdue University undergraduates participated to partially fulfill an introductory psychology course requirement. Participants were run individually in four sessions. Data from four participants were dropped because of error proportions higher than the cutoff. Ages of the eleven remaining participants (two women, nine men) ranged from 18 to 22 years (*M* = 19.36; *SD* = 1.12). Approval for human participants was obtained from the Purdue University Institutional Review Board. The experiment conforms to relevant regulatory standards. Informed consent was obtained from all participants.

***Stimuli and apparatus.*** These were identical to the Single-Task Condition.

***Design and procedure.*** There were two parts in each session: practice at reproducing a time interval, then the list learning and search part.

Time production practice was in the first part of each session. A tone presented the interval, 2400 ms, five times and participants were asked to produce this duration. After these demonstration trials, the target interval was never presented again. The participant was to produce the same interval throughout the session. Each trial began with presentation of asterisks on the screen, indicating the participant could press the middle button to start time production when ready. When the middle button was pressed, a continuous tone was emitted indicating the start of interval production. The participant pressed the left or right button, as the participant wished, to end the tone when it was judged that the target time interval had elapsed. Feedback was given: if the produced interval was within a temporal window of 10% around the target duration (between 2280 and 2520 ms) the feedback was “correct.” Otherwise, the feedback was “too long” or “too short,” as appropriate.

Each of the three blocks of time production practice had 50 time-production trials. The third practice block was identical to the first two, but without feedback.

For the list learning and search part, design was as in the Single-Task Condition, except that in the search phase of a block the participant performed the memory search task concurrently with the time production task. On each trial of the search phase, the tone indicated the beginning of the 2400 ms interval to produce. The participant pressed the left or right button to end the tone when the target interval was judged to have elapsed. Instructions were to press the left button if the probe was from either of the two memory sets and to press the right button if it was absent from both. Each session lasted approximately 60 min.

In the Dual-Task Condition, one participant prematurely pressed the middle button by mistake in the list learning phase. Two blocks were thus invalidated and dropped.

### Results

Reaction times and time productions averaged over trials in which responses were correct are in Table 1; percent errors are in Table 2.

**Table 1:** **Experiment 1 reaction times and time productions**.

	**Memory set**	
**Probe**	**Active**	**Inactive**
**SINGLE-TASK CONDITION**		
Present	829 (181)	881 (179)
Absent	836 (138)	891 (179)
**DUAL-TASK CONDITION**		
Present	3122 (400)	3130 (429)
Absent	3114 (387)	3133 (415)

Standard deviations in parentheses, time in ms.

**Table 2:** **Experiment 1 percent errors**.

	**Memory set**	
**Probe**	**Active**	**Inactive**
**SINGLE-TASK CONDITION**		
Present	5.8 (4.4)	12.5 (5.3)
Absent	3.6 (2.9)	7.6 (2.6)
**DUAL-TASK CONDITION**		
Present	4.2 (2.4)	11.4 (5.6)
Absent	3.9 (2.9)	8.7 (5.0)

Standard deviations in parentheses.

#### Single-task condition

For each session and participant, mean reaction times (RTs) for memory search correct trials and proportion of memory search errors were calculated in each combination of memory set active or inactive, probe present or absent. These means were averaged over sessions and the resulting mean RTs and mean error proportions were input to separate repeated measures ANOVAs with active/inactive and probe presence/absence as factors.

Mean RT was longer and error proportion was larger in the inactive than in the active condition, *F*_(1, 9)_ = 42.36, *p* < 0.001, *MSE* = 688, partial η^2^ = 0.83 and *F*_(1, 9)_ = 51.65, *p* < 0.001, *MSE* = 0.001, partial η^2^ = 0.85, respectively. No other effects or interactions were significant.

There was not a significant effect of whether the probe was present or absent. Some experiments find such an effect on reaction time and some do not (e.g., Sternberg, 1975, Figure 2). Circumstances leading to a significant effect are not well understood.

#### Dual-task condition

ANOVAs of the same form were conducted on time productions (TPs) and proportion of memory search errors. Activating a memory set had increased RT by 54 ms in the Single-Task Condition, but increased TP by only 14 ms, a non-significant effect, *F*_(1, 10)_ = 1.61, n.s., *MSE* = 1225, partial η^2^ = 0.14. Power is high for rejecting at the 0.05 level the null hypothesis of no effect of activation on Time Production in the Dual-Task Condition. It was calculated with G*Power 3 (Faul et al., 2007). For the alternative hypothesis of a small effect (Cohen's *f* = 0.10), power is 0.99996. For the power calculations, the non-sphericity correction ε is 1 and average correlation between repeated measurements in different conditions is 0.995. The power is sensitive to this correlation, which is notably high here, likely because the participant is trying to produce the same time interval every time.

Because the effect of memory set activation is important for the Timing and Complex-Span Hypothesis, we conducted an analysis from a different point of view. We compared two models accounting for the time productions with the Akaike Information Criterion (Akaike, 1974). Briefly, the Akaike Information Criterion (AIC) for a model is *AIC* = −2 ln(*L*) + 2*k*, where *L* is the likelihood and *k* is the number of parameters. The first term is smaller the better the goodness of fit of the model but the second term is larger the more parameters in the model. The AIC integrates a tradeoff between goodness of fit and number of parameters. The numerical value of the AIC is not informative on its own. But a set of models can be compared by selecting the one with smallest AIC. This is not an alternative way of doing a hypothesis test; rather it is a way of selecting the model in the set that is most parsimonious in representing the data.

The full model we considered has all main effects and interactions of the ANOVA that was conducted on time productions. The reduced model we considered omits the main effect of activation and all interactions involving activation. Analysis was done in R with the function lmer in the package lme4 (For discussion of model selection with AIC in R, see Venables and Ripley, 1994). Subjects was a random factor; other factors were fixed. Parameters were estimated with maximum likelihood. The reduced model had smaller AIC, Δ*AIC* = 17.63. We conclude that the more parsimonious model does not include activation or interactions involving it.

Error proportion was higher in the inactive than in the active condition, *F*_(1, 10)_ = 28.56, *p* < 0.001, *MSE* = 0.001, partial η^2^ = 0.74. Other effects were non-significant for TP and errors.

We tested the difference in error proportion in the Single- and Dual-Task Conditions. An ANOVA in the same form as above but with the additional factor condition (Single- and Dual-Task) showed no effect of condition, *F*_(1, 19)_ = 0.063, n.s., *MSE* = 0.003, partial η^2^ = 0.00 (percent errors were 7.38 and 7.05 in the Single- and Dual-Task conditions, respectively), and no interaction between condition and the active/inactive factor, *F*_(1, 19)_ = 0.20, n.s., *MSE* = 0.001, partial η^2^ = 0.01). There was no probe present/absent by condition interaction, *F*_(1, 19)_ = 1.20, n.s., *MSE* = 0.002, partial η^2^ = 0.06.

### Discussion

The objective of Experiment 1 was to test whether activating a memory set from long term memory would interfere with timing. Activation did not have a significant effect on concurrent timing in the Dual-Task Condition. In contrast, activating a memory set from long term memory increased RTs in the Single-Task Condition. Errors did not differ in the Dual- and Single-Task Conditions, showing that the dissociation cannot be explained by a speed-accuracy trade-off. With this paradigm using set size 5, we conclude that timing proceeds in the same way whether the memory set is active or inactive because we see no evidence of activating the memory set on time productions.

The percentage of trials in errors in the search task was relatively high. Averaged over conditions, there were errors in over 7% of the trials (Table 2). Furthermore, data from four participants of fourteen had to be eliminated because their errors exceeded the cutoff of 0.25 proportion of errors in each condition (see Participants section). Despite the difficulty, results are orderly. We see a clear effect of activating a memory set on reaction times, but not on time production.

Note that time productions were generally longer than the target interval to produce. This is a typical finding when time intervals are produced concurrently with other tasks. Our interpretation is the commonly accepted one that time productions are lengthened by general attention demands of a concurrent non-temporal task (Brown, 1997, 2006; Coull et al., 2004). The question of interest is not whether there is a general attention demand from the non-temporal task, but rather whether there is an additional specific demand due to activating a memory set. A lengthening specifically due to activating a memory set was not observed.

In Experiment 1 the memory set always contained five items. In Experiment 2, we tested the effect of varying set size along with the active-inactive manipulation. There were two reasons for investigating these two factors in Experiment 2. The first was to see whether the two factors would have different effects on produced intervals. One factor, increasing set size in memory search, consistently lengthens time intervals produced concurrently (Fortin and Rousseau, 1987; Fortin et al., 2007, 2010; Rattat, 2010). The other factor, activating a memory set, had no effect on concurrent timing in Experiment 1 here. Therefore, we predicted that time productions would lengthen with increasing memory set size, but that memory activation would have no effect.

The second reason for testing jointly set size and activation in Experiment 2 was to see whether the two factors would combine in our Single-Task reaction time condition in the pattern found by Conway and Engle (1994). They found additive effects of the two factors on RT, and additive effects on errors.

## Experiment 2

Conditions were as in Experiment 1 with a few exceptions. Experiment 2 used memory set sizes of 3 and 6. In each block of trials, one memory set was active and one inactive as in Experiment 1. If both sets were of size 3, the total number of items would be 6, and the participant could easily keep both sets active; the task would be equivalent to searching an active set of six items. To discourage this, we used two sizes, three and six, for which the sum exceeds short term memory capacity. Maintaining nine items active in memory being too difficult, we assumed participants would keep the inactive set in its inactive state during a block, activating it only when needed. Set size combinations used were size 3 active with size 6 inactive and size 6 active with size 3 inactive.

### Materials and methods

Method was as in Experiment 1, except memory set size varied, and slight modifications were made in the number of blocks and trials.

#### Single-task condition

***Participants.*** Fourteen Purdue University undergraduates completed the experiment in this condition, to partially fulfill an introductory psychology course requirement. Each was run individually in four sessions. Data from two participants were eliminated because their error proportions were higher than the cutoff. Error proportion was required to be less than 0.25 in each of eight factor combinations (probe present/absent, memory set active/inactive, size of searched memory set 3 or 6), averaged over all four sessions. Ages of the twelve remaining participants (five women, seven men) ranged from 18 to 21 years (*M* = 19.58; *SD* = 1.08). Approval for human participants was obtained from the Purdue University Institutional Review Board. The experiment conforms to relevant regulatory standards. Informed consent was obtained from all participants.

***Design and procedure.*** Each session included nine 21-trial blocks. The first block and the first trial in each block were not analyzed. There were two memory sets for each block: one of three items and another of six items. On half of the eight test blocks, chosen randomly, the word set appeared first for learning. On half of the four blocks in which a word set appeared first, it was selected randomly to be the active memory set. In the two blocks in which the word set appeared first and was also selected as the active memory set, the word set for one block consisted of six items and that for the letters consisted of three. The same applied for the four blocks on which the letter set appeared first. On the first practice block, the memory set presented first, the active memory set, and the set size of the memory sets were all chosen randomly.

On the first trial of each block, the probe was selected as described in Experiment 1. On half the remaining 20 trials, the probe was from the active pool and on half from the inactive pool, randomly. On half of the 10 trials with a probe from the active pool, the probe was present in the memory set and on half it was absent, randomly. The same applied for the 10 trials with the probe from the inactive pool. Each session lasted approximately 40 min.

#### Dual-task condition

***Participants.*** Twenty Purdue undergraduates completed this condition to partially fulfill an introductory psychology course requirement. Data from five were dropped because error proportions were higher than the cutoff. Ages of the fifteen remaining participants (four women, eleven men) ranged from 18 to 23 (*M* = 19.33; *SD* = 1.45). Approval for human participants was obtained from the Purdue University Institutional Review Board. The experiment conforms to relevant regulatory standards. Informed consent was obtained from all participants.

***Design and procedure.*** Design was as for the Dual-Task Condition of Experiment 1, but with set sizes and number of blocks and trials as in the Single-Task Condition above. Each session lasted approximately 60 min.

### Results

Occasionally a participant pressed the middle button prematurely in the list learning phase, invalidating a block of trials. In the Single-Task Condition, two such blocks were dropped for one participant and one for another. In the Dual-Task Condition, two such blocks were dropped for one participant.

Table 3 shows RTs and TPs averaged over trials in which responses in the search task were correct, with percent errors in memory search in Table 4. ANOVAs were performed as in Experiment 1, with Set Size an additional factor crossed with the other factors. Four separate ANOVAs were carried out, on RTs and error proportion in the Single-Task Condition, and on TPs and error proportion in the Dual-Task Condition.

**Table 3:** **Experiment 2 reaction times and time productions**.

	**Memory Set**			
	**Active**	**Active**	**Inactive**	**Inactive**
**Probe**	**Size 3**	**Size 6**	**Size 3**	**Size 6**
**SINGLE-TASK CONDITION**				
Present	830 (166)	933 (292)	921 (224)	1003 (309)
Absent	880 (189)	969 (263)	928 (217)	1016 (258)
**DUAL-TASK CONDITION**				
Present	3419 (853)	3530 (969)	3530 (798)	3463 (889)
Absent	3418 (855)	3548 (992)	3530 (993)	3469 (894)

Standard deviations in parentheses, time in ms.

**Table 4:** **Experiment 2 percent errors**.

	**Memory Set**			
	**Active**	**Active**	**Inactive**	**Inactive**
**Probe**	**Size 3**	**Size 6**	**Size 3**	**Size 6**
**SINGLE-TASK CONDITION**				
Present	3.5 (2.7)	6.2 (3.9)	10.7 (6.4)	12.6 (5.5)
Absent	1.1 (1.9)	2.8 (3.3)	3.7 (2.9)	7.0 (4.6)
**DUAL-TASK CONDITION**				
Present	3.6 (2.9)	8.0 (4.9)	13.9 (6.2)	13.7 (6.5)
Absent	1.8 (2.0)	4.4 (3.2)	7.0 (4.5)	10.9 (7.0)

Standard deviations in parentheses.

#### Single-task condition

RTs were longer in the inactive than in the active condition, *F*_(1, 11)_ = 11.08, *p* < 0.01, *MSE* = 8885, partial η^2^ = 0.50, and longer at set size 6 than 3, *F*_(1, 11)_ = 13.44, *p* < 0.01, *MSE* = 14,700, partial η^2^ = 0.55. The interaction between Set Size and Active/Inactive was not significant, *F*_(1, 11)_ = 0.21, n.s., *MSE* = 3738, partial η^2^ = 0.02.

The combined effect of Set Size and Active/Inactive is important because the model in which memory set activation precedes memory set search predicts additive effects of these factors on reaction time. We used the AIC to compare the full model for reaction time that has all main effects and interactions of the ANOVA previously conducted with a reduced model that omits the interaction of Set Size and Active/Inactive and all higher order interactions involving both factors. Analysis was done in R with the function lmer in the package lme4. Subjects was a random factor; other factors were fixed. Parameters were estimated with maximum likelihood. The reduced model had smaller AIC, Δ AIC = 29.85. We conclude that the more parsimonious model does not include interactions involving set size and activation.

Error proportion was higher in the inactive than in active condition, *F*_(1, 11)_ = 30.30, *p* < 0.001, *MSE* = 0.002, partial η^2^ = 0.73, and higher at size 6 than 3, *F*_(1, 11)_ = 7.64, *p* < 0.05, *MSE* = 0.002, partial η^2^ = 0.41. For errors, the interaction between active/inactive and set size was not significant, *F*_(1, 11)_ = 0.17, n.s., *MSE* < 0.001, partial η^2^ = 0.02. Error proportion was higher when the probe was present than when it was absent, *F*_(1, 11)_ = 19.69, *p* < 0.01, *MSE* = 0.003, partial η^2^ = 0.64. Other effects were non-significant.

#### Dual-task condition

For TPs, the main effect of active/inactive was not significant, *F*_(1, 14)_ = 1.99, n.s., *MSE* = 5459, partial η^2^ = 0.12, but TPs were longer at set size 6 than at size 3, *F*_(1, 14)_ = 11.96, *p* < 0.01, *MSE* = 2015, partial η^2^ = 0.46.

The interaction between active/inactive and set size was significant, *F*_(1, 14)_ = 7.93, *p* < 0.05, *MSE* = 32,089, partial η^2^ = 0.36. The interaction is hard to interpret. To obtain details, simple main effects were tested. For set size 3, TPs were significantly longer for the inactive memory set, *F*_(1, 14)_ = 8.04, *p* = 0.013, partial η^2^ = 0.37. Mean TPs were 3419 ms and 3530 ms for active and inactive memory sets, respectively. For set size 6, TPs were significantly longer for the active memory set, *F*_(1, 14)_ = 5.52, *p* = 0.034, partial η^2^ = 0.28. Mean TPs were 3539 ms and 3466 ms for active and inactive memory sets, respectively. Simple main effects of set size were also tested. When an active memory set was searched, TPs were significantly longer for set size 6, *F*_(1, 14)_ = 10.90, *p* = 0.005, partial η^2^ = 0.44. When an inactive memory set was searched, set size was not significant, *F*_(1, 14)_ = 4.31, *p* = 0.057, partial η^2^ = 0.24. (In case a correction for the number of *post hoc* tests is desired, *p* values are reported). The simple main effects of memory set activation are contrary to the Timing and Complex-Span Hypothesis, but it is hard to understand why the effect would go in opposite directions for different set sizes.

Error proportion was higher in the inactive than in the active condition, *F*_(1, 14)_ = 73.60, *p* < 0.001, *MSE* = 0.002, partial η^2^ = 0.84; at set size 6 than at set size 3, *F*_(1, 14)_ = 12.28, *p* < 0.01, *MSE* = 0.002, partial η^2^ = 0.47; and when the probe was present rather than absent, *F*_(1, 14)_ = 21.45, *p* < 0.001, *MSE* = 0.002, partial η^2^ = 0.61. No two-way interactions were significant. The three-way interaction was significant [*F*_(1, 14)_ = 7.65, *p* < 0.05, *MSE* = 0.001, partial η^2^ = 0.35]. The three-way interaction has the following form. In Table 4, Dual-Task Condition, errors are always higher for probe present than absent, always higher for inactive memory set than active, and higher for set size 6 than 3 except for the single case of probe present, inactive memory set.

If processing in the memory task were done the same way in the Single- and Dual-Task conditions, error proportions would be comparable. To test this, an ANOVA was performed on the error proportions combining the two conditions. The ANOVA had form as those above, but with the additional factor of Condition (Single- vs. Dual-Task) crossed with the other factors. Condition was not significant, *F*_(1, 25)_ = 3.21, n.s., *MSE* = 0.006, partial η^2^ = 0.11. There was no significant interaction of Condition with any other factor or combination of factors. As far as one can determine from errors, processing the memory set was performed the same way in both conditions.

The main results expected in Experiment 2 were that (1) memory activation and increased set size would increase RTs in the Single-Task Condition, and that (2) time productions would lengthen with set size, but not with memory activation in the Dual-Task Condition. As expected, RTs increased with set size and were longer with memory activation. Even though on average time productions lengthened with set size and did not differ in the active and inactive conditions, an interaction was observed, showing opposite effects of activation for different set sizes. Before discussing this puzzling result, we consider the second objective of Experiment 2. Specifically, effects of activation and set size will be examined to test process organization.

#### Process organization

In the model of Wickens et al. (1981), Wickens et al. (1985), and Conway and Engle (1994), the memory set is activated and then it is searched (see lower part of Figure 1). If two factors selectively influence two processes in series, the factors are predicted to have additive effects on reaction time (Sternberg, 1969). It is sometimes thought that for the Additive Factor Method to apply, errors must be the same in all conditions, or responses must be speeded, but such stringent conditions are not needed (Schweickert, 1985; Schweickert et al., 2012). As the model predicts, the two factors, active/inactive memory set and set size, have significant and additive effects on reaction time in the Single-Task Condition. (Probe presence/absence had no effect on reaction time). An analogous non-significant interaction was also found by Wickens et al. (1981), Wickens et al. (1985) and Conway and Engle (1994), set size two sometimes an exception in the last study. These results support the serial organization of activation and search.

Conway and Engle (1994) also reported that factors influencing activation and search had additive effects on error probability. This can be explained with the same serial process organization (Schweickert, 1985; Schweickert et al., 2012). Suppose the probability of a correct response equals.

P[Correctly Activate Memory Set]

× P[Correctly Search|Correctly Activate Memory Set].

Now suppose one factor changes the probability of correctly activating the memory set, and another factor changes the probability of correctly searching, given correct activation of the memory set, each factor changing only one probability. Then the combined effect of the two factors on probability correct is the product of their individual effects. Multiplicative effects on probability correct could have been manifest as additive, through the following approximation. Multiplicative effects on probability correct correspond to additive effects on the logarithm of probability correct. But the natural log of a relatively large probability *P* is approximately equal to −(1 − *P*). For example, log 0.95 = −0.051. Suppose one process is correct with probability *p*, another is correct with probability *q*, and the probability of a correct response is *r* = *pq*. If one factor changes *p* and another factor changes *q* the factors will have multiplicative effects on probability of a correct response. If *r* = *pq*, then log *r* = log *p* + log *r*. A little algebra shows that if the probabilities are relatively large, the multiplicative effects predict approximately additive effects.

A model in which active/inactive memory set and set size have multiplicative effects was fit to frequencies of correct responses; see Appendix A. Predicted and observed values are quite close in both the Single-Task and Dual-Task Conditions (Table 5).

**Table 5:** **Observed frequencies of responses and predictions**.

**Memory set**	**Response**	**Set size**			
		**3**		**6**	
		**Obs**	**Pred**	**Obs**	**Pred**
**SINGLE-TASK CONDITION**					
Active	Correct	1817	1818.28	1863	1860.23
Inactive	Correct	1810	1805.64	1675	1680.71
Active	Incorrect	43	41.72	87	89.77
Inactive	Incorrect	140	144.36	185	179.29
**DUAL-TASK CONDITION**					
Active	Correct	2316	2312.33	2251	2258.61
Inactive	Correct	2149	2161.81	2093	2076.54
Active	Incorrect	64	67.67	149	141.40
Inactive	Incorrect	251	238.19	287	303.46

If Active/Inactive Memory Set and Set Size Have Multiplicative Effects in Experiment 2.

For comparison, a model in which the two factors have additive effects was also fit. For both models the goodness-of-fit statistic, *G*^2^, has approximately a chi-square distribution with 1 *df*. In the Single-Task condition, for the multiplicative model *G*^2^ = 0.47 and for the additive model *G*^2^ = 0.32. The small values of *G*^2^ indicate that both models fit very well. Parameters were estimated to minimize *G*^2^, so the AIC for a model equals *G*^2^ plus the number of parameters (see, e.g., Moshagen, 2010). The number of parameters is the same, 3, for each model. The additive model has slightly smaller AIC, Δ*AIC* = 0.15. The additive model is more parsimonious, but negligibly so.

In the Dual-Task condition, for the multiplicative model *G*^2^ = 2.43 and for the additive model *G*^2^ = 8.54. The multiplicative model has smaller AIC,Δ*AIC* = 6.11. The multiplicative model fits well and is more parsimonious than the additive model.

Reaction times in the Single-Task Condition and accuracy in both the Single- and Dual-Task conditions are all consistent with the process organization of activation preceding search. (The order of these two processes is not established, but it seems more natural for activation to precede search than the reverse).

Two objections to the multiplicative model for accuracy may be raised. First, the model is fit to averages over participants. But the average of a product does not equal the product of the average of the multiplicands, if the multiplicands are correlated. In response, we note that in our data the correlations are low or moderate. For proportion correct the average correlation between repeated measures across combinations of factor levels is 0.11 in the Single-Task Condition and 0.41 in the Dual-Task Condition. A second objection is that with a multiplicative model for accuracy factor effects are not additive, but significant interactions were not found between set size and activation in the ANOVAs. Further, in the Single-Task Condition, a multinomial tree model with additive effects is more parsimonious (albeit barely) than a multiplicative model. In response we note that the sizes of the interactions predicted by the multiplicative model are quite small for proportion correct: 0.001 for the Single Task Condition and 0.002 for the Dual Task Condition. With such small interactions predicted by the multiplicative model, it is not surprising that an additive model can perform well. Further, a multiplicative model has a natural interpretation: the probability of a correct response is the probability of correct activation followed by correct search given the correct activation.

### Discussion

Experiment 2 was informative about our secondary objective, to test the model in which memory set activation precedes search. In the Single-Task Condition, effects on RT of memory set activation and set size were additive, supporting the model. The model was further supported because the equation in which the factors have multiplicative effects on proportion correct fit well. In the Dual-Task Condition, the multiplicative equation also fit effects on proportion correct well.

Information from Experiment 2 about our primary objective, testing whether memory set activation interferes with timing, is complicated by an interaction on TPs between memory set activation and set size.

#### A puzzling result

For time productions, it is hard to interpret the interaction of memory set activation and set size. When an inactive memory set was searched, it is peculiar that produced intervals were numerically shorter when set size was 6 than when it was 3. The direction is surprising because in the Single-Task Condition, reaction times were significantly longer with higher set size. Increasing set size has consistently lengthened produced intervals previously (e.g., Fortin and Rousseau, 1987; Neath and Fortin, 2005; Fortin et al., 2007). The interaction may have something to do with the high difficulty level of the task. Participants had to memorize two new memory sets on each block. High error proportions led to dropping data from several participants. Difficulty may have led participants to terminate temporal productions too quickly in the inactive memory set condition, when set size was 6.

To pursue this puzzling result, one would want to investigate a wider range of memory set sizes. But using a range of set sizes is not feasible with the paradigm of Experiments 1 and 2. A participant learns two memory sets and the active one is presented again at the start of every trial. The active and inactive memory sets are treated differently, so using more than two set sizes would require considerable counterbalancing. A paradigm treating memory sets more symmetrically would be more suitable.

The paradigm of the two first experiments has been useful. It allowed us to observe different effects for active and inactive memory sets, with systematic effects on reaction time and accuracy data. A clear dissociation was found in Experiment 1, with longer reaction times in the inactive condition, but unchanged time productions. In Experiment 2, evidence from reaction times and errors was consistent with the model in which the two factors selectively influenced sequential processes. However, error proportions were high, time productions in Experiment 2 showed an unusual shortening of produced intervals with increasing set size in the inactive memory set condition, and the paradigm cannot be efficiently used for testing more than two memory set sizes. For these reasons, a related but different paradigm of Conway and Engle (1994) was used in Experiment 3.

## Experiment 3

Experiment 3 tested the effect of activating information in memory on concurrent timing, using a memory task of Conway and Engle (1994, Experiment 4). The participant memorized four memory sets, to a stricter criterion than in our Experiments 1 and 2. Three factors varied: the delay between a cue indicating which set to search and a probe, set size, and presence/absence of the probe in the set. A delay between cue and probe allowed the participant time to activate the appropriate memory set in advance, so it could be searched immediately when the probe appeared. On a trial with no delay, the participant presumably had to activate the memory set in order to search it when the probe appeared.

### Materials and methods

Method is as in Experiments 1 and 2 with exceptions described below.

#### Single-task condition

***Participants.*** Ten participants (four men, six women), between 18 and 41 years old (*M* = 24.8; *SD* = 6.68) and recruited through advertisement at Laval University, were given a C$5 honorarium for one session of about 50 min. Approval for human participants was obtained from the Human Research Ethics Committee at Laval University (CÉRUL). The experiment conforms to relevant regulatory standards. Informed consent was obtained from all participants.

***Stimuli and apparatus.*** Responses used the three leftmost keys of a five-key response box. Visual stimuli were presented on a 17 in. monitor about 70 cm in front of the participant. They were in white letters on a black background, Courier New, font size 18 points.

The word pool was 42 four-letter French words (Appendix B), from the high-frequency words in the OMNILEX database (University of Ottawa). Word sets included 3, 4, 5, or 6 different words. For each participant, 18 words were selected randomly from the word pool and assigned randomly to the four sets. A digit, “3,” “4,” “5,” or “6” was presented with each set, the number of words in the set. Each set was presented in a vertical column, centered on the screen, below the digit. Sets of 3, 4, 5, and 6 items were displayed for 40, 50, 60, and 70 s respectively.

***Design and procedure.*** In the list-learning phase, the four sets were successively presented in random order. After two presentations of the four sets, the experimenter asked the participants to recall each, identifying the sets with their digits (e.g.: “Please recall the words in the list containing four words.”). Recall was correct when the participant recalled the words (in any order) in the set three times successively. The sets were recalled in random order, but the last studied set was never presented first for recall. After testing the four sets, sets with mistaken recall were presented anew, until all sets were recalled three times successively with no error.

The list-search phase then began with a last successive presentation of all sets. Pressing the middle button started the trial with “+,” displayed for 1 s, then replaced by a digit above a probe centered on the screen. The digit could be presented simultaneously with the probe (no-delay) or one second before the probe (delay). Participants were asked to indicate as quickly as possible whether the probe word was or not in the list identified by the digit by pressing a left or right key on the response box. One second after the response, the next trial began with “+.” There was a single block of 192 trials with no pause.

Set size, delay, and probe presence/absence were determined randomly on each trial, with levels of these factors balanced in the block. If the probe was absent from the set to be searched, it was member of one of the other three sets.

#### Dual-task condition

***Participants.*** Thirteen participants (five men, eight women), between 21 and 38 years old (*M* = 24.5; *SD* = 4.86) completed one session of about 50 min. Approval for human participants was obtained from the Human Research Ethics Committee at Laval University (CÉRUL). The experiment conforms to relevant regulatory standards. Informed consent was obtained from all participants.

***Design and procedure.*** Design was as in the Single-Task Condition except that participants were asked to respond to the probe when the tone duration had reached the previously learned target interval (2400 ms).

### Results and discussion

RTs and TPs averaged over correct memory search responses are in Table 6 and percent errors in Table 7.

**Table 6:** **Experiment 3 reaction times and time productions**.

	**Memory Set**			
**Probe**	**Size 3**	**Size 4**	**Size 5**	**Size 6**
**SINGLE-TASK CONDITION**				
**No delay**				
Present	1107 (231)	1377 (273)	1437 (451)	1321 (283)
Absent	1360 (276)	1587 (335)	1546 (308)	1479 (298)
**Delay**				
Present	868 (257)	1099 (349)	1204 (439)	1137 (277)
Absent	1015 (330)	1188 (286)	1410 (251)	1204 (251)
**DUAL-TASK CONDITION**				
**No delay**				
Present	3460 (1502)	3488 (1346)	3497 (1389)	3589 (1412)
Absent	3511 (1305)	3634 (1289)	3663 (1479)	3659 (1372)
**Delay**				
Present	3379 (1483)	3519 (1465)	3594 (1460)	3468 (1307)
Absent	3478 (1351)	3656 (1596)	3576 (1392)	3638 (1445)

Standard deviations in parentheses, time in ms.

**Table 7:** **Experiment 3 percent errors**.

	**Memory Set**			
**Probe**	**Size 3**	**Size 4**	**Size 5**	**Size 6**
**SINGLE-TASK CONDITION**				
**No delay**				
Present	1.7 (3.5)	2.5 (5.6)	1.7 (3.5)	3.3 (4.3)
Absent	2.5 (4.0)	3.3 (4.3)	2.5 (4.0)	2.5 (4.0)
**Delay**				
Present	0.8 (2.6)	0.8 (2.6)	5.0 (7.0)	4.2 (5.9)
Absent	0.8 (2.6)	3.3 (5.8)	0.8 (2.6)	1.7 (3.5)
**DUAL-TASK CONDITION**				
**No delay**				
Present	1.9 (3.7)	3.2 (5.4)	5.1 (6.4)	6.4 (8.4)
Absent	1.9 (5.0)	3.2 (9.3)	8.3 (17.0)	2.6 (5.3)
**Delay**				
Present	2.6 (7.1)	4.5 (6.5)	3.2 (5.4)	5.1 (8.0)
Absent	0.6 (2.3)	2.6 (6.3)	9.6 (13.5)	2.6 (4.0)

Standard deviations in parentheses.

#### Single-task condition

Mean RTs in correct trials and error proportions were calculated at each combination of delay (no-delay, 1 s delay), memory set size (3, 4, 5, or 6 items) and probe (present, absent) factors. These means were input to separate repeated-measure ANOVAs on RTs and error proportions.

RTs were longer in the no-delay than in the delay condition, *F*_(1, 9)_ = 61.19, *p* < 0.001, *MSE* = 41,852, partial η^2^ = 0.88. RTs were 261 ms longer in the no-delay condition, which we interpret as the time required to activate the identified memory set. We note that the effect of activation is larger in this experiment than in the previous two. Wickens et al. (1985) noted different sizes of the effect of activation in their two experiments. In our case the difference may be due to four sets in long term memory in Experiment 3, more than in our previous two experiments. RTs were longer in probe-absent trials, *F*_(1, 9)_ = 25.77, *p* < 0.01, *MSE* = 37,261, partial η^2^ = 0.74. RTs changed with increasing set size, *F*_(3, 27)_ = 12.86, *p* < 0.001, *MSE* = 53,949, partial η^2^ = 0.59. In Table 6 RT tends to increase with Set Size, but drops at set size 6. There is a significant linear trend, *F*_(1, 9)_ = 30.64, *p* < 0.001, *MSE* = 30187, partial η^2^ = 0.77. But there is also a significant quadratic trend, *F*_(1, 9)_ = 14.29, *p* < 0.01, partial η^2^ = 0.61. We do not know why the decline at highest set size occurs, but it also occurred in three of the four experiments that used this paradigm in Conway and Engle (1994, Figures 3, 4, 6).

No interactions were significant, including that between delay and set size, *F*_(3, 27)_ = 1.49, n.s., *MSE* = 31,021, partial η^2^ = 0.14. The non-significant interaction between delay and set size is consistent with the model used by Wickens et al. (1981), Wickens et al. (1985), and Conway and Engle (1994), in which the memory set is activated and then searched.

The combined effect of set size and active/inactive is important because the model in which memory set activation precedes memory set search predicts additive effects of these factors on RT. We used the AIC to compare the full model for RT, which has all main effects and interactions of the ANOVA previously conducted, with a reduced model, which omits the interaction of set size and active/inactive and all higher order interactions involving both factors. Analysis was done in R with function lmer in package lme4. Subjects was a random factor; other factors were fixed. Parameters were estimated with maximum likelihood. The reduced model had smaller AIC, Δ*AIC* = 15.19. We conclude that the more parsimonious model does not include interactions involving set size and activation.

The ANOVA on error proportion showed no effect of delay, set size or probe presence/absence, nor any interaction.

### Dual-task condition

Effects on time productions of memory search are markedly different from those of memory activation (Table 6). The increase in TPs produced by increasing memory set size from three to six, 131 ms, is about two-thirds of the increase produced on RT, 198 ms. On the other hand, the increase in TPs produced by activating a memory set, 24 ms, is less than a tenth of the increase produced on RT, 261 ms.

ANOVAs as in the Single-Task Condition were performed in Dual-Task Condition. Set size had a significant effect on TPs, *F*_(3, 36)_ = 5.63, *p* < 0.01, *MSE* = 36,318, partial η^2^ = 0.32, an effect consistently observed (Fortin and Rousseau, 1987; Fortin et al., 2007; Rattat, 2010), and interpreted to mean timing slows or pauses when an active memory set is searched. In Table 6, TPs tend to numerically increase with set size, with a leveling off or decline at the highest set size. There is a significant linear trend of TP with set size, *F*_(1, 12)_ = 15.71, *p* < 0.01, *MSE* = 26,890, partial η^2^ = 0.57. The quadratic trend is not significant, but nearly so, *F*_(1, 12)_ = 4.19, *p* = 0.06, *MSE* = 38,556, partial η^2^ = 0.26. The pattern is like that of the reaction times.

TPs were longer in probe-absent than in probe-present trials, *F*_(1, 12)_ = 7.47, *p* < 0.05, *MSE* = 73,507, partial η^2^ = 0.38, an effect sometimes observed when memory search is performed concurrently with time production (e.g., Fortin and Rousseau, 1987).

However, the factor corresponding to memory set activation, delay, had no significant effect on TPs, *F*_(1, 12)_ = 0.64, n. s., *MSE* = 47,166, partial η^2^ = 0.05. Power for rejecting at the 0.05 level the null hypothesis of no effect of activation on TPs was calculated with G^*^power3 (Faul et al., 2007). If the true effect is small (Cohen's *f* = 0.10), the power is 0.84. (For power calculation, the average correlation between repeated measurements is 0.983, the non-sphericity correction ε is 1). Power is sensitive to the correlation between repeated measurements, which is high here.

Because the effect of activation on time production is important for the Timing and Complex-Span Hypothesis, we compare the full model, having all main effects and interactions of the ANOVA previously done, with a reduced model omitting activation and all higher order interactions involving activation. Analysis was done in R with function lmer in package lme4. Subjects was a random factor, other factors were fixed. Parameters were estimated with maximum likelihood. The reduced model had smaller AIC, Δ*AIC* = 47.92. We conclude that the more parsimonious model does not include activation or interactions involving it.

There were no other significant effects in this analysis. There were no significant effects in the ANOVA on error proportions.

In this paradigm, activating a memory set and searching the memory set both take time and presumably involve short-term memory. Nonetheless, only memory search interferes with timing.

## General discussion

Participants reproduced a time interval concurrently with performing a memory search task. We tested whether activating the memory set interferes with timing. No interference was found in Experiment 1. In Experiment 2, where memory activation was manipulated jointly with memory set size, timing results showed unexpected and opposite effects of memory activation in the low and high memory load conditions. Error data suggested that this complex pattern of disruption of timing may have been produced by extreme difficulty in processing the inactive memory set. This led us to use a related but different paradigm in Experiment 3. This paradigm, borrowed from Conway and Engle (1994), resulted in lower error proportions. Predictions regarding timing productions were confirmed under those conditions: no interference due to activation, while in contrast, time productions lengthened with increasing set size (as in Fortin and Rousseau, 1987 and Fortin et al., 2007). Results overall favor the conclusion that activating a memory set does not interfere with concurrent timing. Results from Experiment 2 suggest this statement must be restricted to conditions where memory search difficulty is moderate. Overall, results support the Timing and Complex-Span Hypothesis, because performance on activating a memory set is not related to Complex-Span (Conway and Engle, 1994).

An interpretation of the time production interference due to memory search in terms of a widely used accumulation model of timing (Gibbon et al., 1984; Zakay and Block, 1996; Brown, 2006; Buhusi and Meck, 2009) is that searching a memory set interferes with at least one timing mechanism requiring attention or memory. In the present study, a target interval is presented to the participant, who estimates its duration and stores it in memory. The model assumes that when the interval is presented pulses are emitted by a “pacemaker” and accumulated. This process requires constant attention. An attention-controlled gate (Zakay and Block, 1996) or switch (Gibbon et al., 1984, see Lejeune, 1998) allows transfer of temporal information from the pacemaker to the accumulator if attention is devoted to time. When the interval ends, the output of the accumulator may be stored in working memory and then transferred to long-term memory in the form of a criterion, a pulse count that will be used later when producing the target interval. In experimental trials, production begins with a key press, and pulses must again be accumulated until the criterion is reached. Accumulation again requires continuous attention, and the production is ended with a second key press when it is judged that the accumulated pulse count corresponds to the criterion. At any moment during accumulation, the current accumulated count must be continuously compared to the criterion in long-term memory.

Attention is critical to hold the gate (or switch) to the accumulator so pulses are accumulated, and memory is required to store output from the accumulator, to store the criterion, and to compare the output with the criterion. Increase of produced intervals with increasing memory set size suggests that searching short-term memory disrupts the accumulation process, delaying the time when the criterion is reached. However, in Experiments 1 and 3 activating a memory set that is in long-term memory did not interfere with any of the timing mechanisms. This suggests that those mechanisms requiring attention or memory are involved in memory search but not in memory activation.

A result in the previous literature on memory search is contrary to the Timing and Complex-Span Hypothesis that a process interferes with concurrent timing if and only if process performance is related to complex span. Because the result is evidence against the hypothesis we propose, we describe the details. The relation between complex span and short-term memory search was investigated by Conway and Engle (1994). Their paradigm for memory search was described here in the introduction to Experiment 3. In their Experiments 1 and 2 the four memory sets to be searched had items in common, while in Experiments 3 and 4 the four memory sets were pairwise mutually exclusive, a difference that lead to different results. In their Experiments 1 and 2, the effect of set size on RT was greater for low- than high-complex-span individuals on target present trials. This supports the Timing and Complex-Span Hypothesis.

In their Experiments 3 and 4, however, there was no interaction between span and set size on RT or errors. This result is contrary to our hypothesis because in Experiment 3 here, we found greater interference in timing with larger memory sets, despite the memory sets being pairwise mutually exclusive.

From the difference between their first two experiments and their last two, Conway and Engle (1994) concluded that complex-span is related to search of a short-term memory set only when there is competition among the memory sets that are possibly relevant; during search of short-term memory, the executive component of working memory capacity is needed only to inhibit irrelevant information.

If we pursue this reasoning for timing, the reason short-term memory search interferes with concurrent timing when memory sets do not overlap is not because of demand for the executive component. By elimination, the interference must be due to the demand for short-term memory capacity.

The lack of interaction between complex-span and memory set size in Experiments 3 and 4 of Conway and Engle (1994), if it is replicable, requires a modification of our hypothesis. A suitable revision is that if performance of a process is related to complex-span, then the process interferes with concurrent timing.

### Organization of activation and memory search

According to a model proposed by Wickens et al. (1981) memory set activation is carried out before memory set search. The model predicts that a factor selectively influencing activation and a factor selectively influencing search would have additive effects on reaction time (Sternberg, 1969). Non-significant interactions found by Wickens et al. (1981), Wickens et al. (1985), and Conway and Engle (1994) support the model, as do non-significant interactions here in Experiments 2 and 3.

The model also predicts two such factors to have multiplicative effects on the probability of a correct response (Schweickert, 1985). Here a multiplicative model fit accuracy data from Experiment 2 well. (Multiplicative effects were not tested in Experiment 3 because in neither the Single-Task nor Dual-Task Condition did both factors have a significant effect on errors). Conway and Engle (1994) report set size and delay as having additive effects on error probability. We interpret such additivity as occurring because when probability of a correct response is high, multiplicative effects are approximately additive (Schweickert, 1985; Schweickert et al., 2012).

Activation is like moving a pointer to the memory set. Reaction time and accuracy results reveal a clear distinction between activation of a memory set and searching that memory set, and time production results reveal a clear dissociation between them.

### Conclusions

Experiments here on memory set activation support on the whole the Timing and Complex-Span Hypothesis that a process interferes with concurrent timing if and only if process performance is related to complex-span. A result of theoretical importance contrary to the Hypothesis is from Experiments 3 and 4 of Conway and Engle (1994), see the General Discussion. For process organization, data here are consistent with the model of Wickens et al. (1981) in which a memory set is in long term memory is activated and then searched. Our results suggest that when ongoing internal events, such as mental time travel, produce cues that activate information in memory, concurrent timing and complex span are not affected unless the activated information is used.

### Conflict of interest statement

The authors declare that the research was conducted in the absence of any commercial or financial relationships that could be construed as a potential conflict of interest.

## Appendix A

Experiment 2 Model Fitting: Activating a Memory Set and Its Set Size Have Multiplicative Effects on Probability Correct

Let *p_ij_* be the probability of a correct response when the factor active/inactive memory set is at level *i* and the factor set size is at *j* items. The multiplicative model predicts

$$p_{ij}=a_{i}s_{j},$$

where *a_i_* is the probability the memory set is activated correctly and *s_j_* is the probability the search is carried out correctly. Parameters were estimated with Excel Solver to minimize log likelihood, *G*^2^, (e.g., Bishop et al, 1975). Because parameter values are not uniquely determined, the probability the active memory set was activated correctly was arbitrarily set to *a*_1_ = 0.999999.

With this setting, other parameter values are uniquely determined.

For the Single-Task Condition, estimated parameters were *a*_2_ = 0.947181 (inactive memory set activated), *s*_3_ = 0.977595, and *s*_6_ = 0.953990. For the Dual-Task Condition, estimated parameters were *a*_2_ = 0.927115 (inactive memory set activated), *s*_3_ = 0.971567, and *s*_6_ = 0.941086. Predicted frequencies were calculated with these parameters (Table 5).

## Appendix B

Experiment 3 Memory Set Pool

Midi, Papa, Faim, Jour, Lait, Cinq, Rien, Tête, Auto, Main, Café, Gars, Pain, Bain, Loin, Soif, Prix, Ciel, Film, Sept, Idée, Hier, Prof, Reçu, Aide, Bien, Mère, Fête, Bras, Bébé, Dire, Noir, Fait, Test, Soir, Sexe, Côté, Bleu, Pied, Gens, Date, Mâle
